# Evaluation of pea genotype PI180693 partial resistance towards aphanomyces root rot in commercial pea breeding

**DOI:** 10.3389/fpls.2023.1114408

**Published:** 2023-03-14

**Authors:** Carol Kälin, Agnese Kolodinska Brantestam, Anna-Kerstin Arvidsson, Mukesh Dubey, Malin Elfstrand, Magnus Karlsson

**Affiliations:** ^1^ Department of Forest Mycology and Plant Pathology, Swedish University of Agricultural Sciences, Uppsala, Sweden; ^2^ Nomad Foods Ltd., Findus Sverige AB, Bjuv, Sweden

**Keywords:** *Aphanomyces euteiches*, pea root rot, *Phytophthora pisi*, resistance, breeding

## Abstract

The cultivation of vining pea (*Pisum sativum*) faces a major constraint with root rot diseases, caused by a complex of soil-borne pathogens including the oomycetes *Aphanomyces euteiches* and *Phytophtora pisi*. Disease resistant commercial varieties are lacking but the landrace PI180693 is used as a source of partial resistance in ongoing pea breeding programs. In this study, the level of resistance and their interaction with *A. euteiches* virulence levels of six new back-crossed pea breeding lines, deriving from the cross between the susceptible commercial cultivar Linnea and PI180693, were evaluated for their resistance towards aphanomyces root rot in growth chamber and green house tests. Resistance towards mixed infections by *A. euteiches* and *P. pisi* and commercial production traits were evaluated in field trials. In growth chamber trials, pathogen virulence levels had a significant effect on plant resistance, as resistance was more consistent against *A. euteiches* strains exhibiting high or intermediate virulence compared with lowly virulent strains. In fact, line Z1701-1 showed to be significantly more resistant than both parents when inoculated with a lowly virulent strain. In two separate field trials in 2020, all six breeding lines performed equally well as the resistant parent PI180693 at sites only containing *A. euteiches*, as there were no differences in disease index. In mixed infections, PI180693 exhibited significantly lower disease index scores than Linnea. However, breeding lines displayed higher disease index scores compared with PI180693, indicating higher susceptibility towards *P. pisi*. Data on seedling emergence from the same field trials suggested that PI180693 was particularly sensitive towards seed decay/damping off disease caused by *P. pisi*. Furthermore, the breeding lines performed equally well as Linnea in traits important for green pea production, again emphasizing the commercial potential. In summary, we show that the resistance from PI180693 interacts with virulence levels of the pathogen *A. euteiches* and is less effective towards root rot caused by *P. pisi*. Our results show the potential use of combining PI180693 partial resistance against aphanomyces root rot with commercially favorable breeding traits in commercial breeding programs.

## 1 Introduction

The production of pea (*Pisum sativum* L.) is globally on the rise as the easy-to-grow crop poses an important source for food and feed (https://www.fao.org). Peas are widely cultivated as an environmentally sustainable alternative to soybean in many plant-based products, due to their high nutritional value and protein content (Xiong et al., 2018; Wei et al., 2020). *P. sativum* can be grown worldwide in temperate to cool climates with Sweden being one of the northernmost regions of pea cultivation. In Sweden, different pea cultivars have been grown since Neolithic times and the plant has remained one of the country’s most important crop species alongside cereals (Osvald, 1959; Hjelmqvist, 1979; Leino et al., 2013).

Root rot, a soil-borne disease caused by a complex of fungal and oomycete pathogens, poses a major threat to commercial pea production. Oomycetes resemble fungi in morphology and growth but are able to reproduce both asexually *via* motile zoospores and with the production of sexual oospores. The oospores are resilient to desiccation and can remain in the soil as inoculum for several years (Mitchell and Yang, 1966; Cannesan et al., 2011). Among these root rot pathogens, *Aphanomyces euteiches* is the main causal agent for pea root rot. Its symptoms include discoloration of roots and epicotyl, root damage, wilting and eventual severe yield losses (Malvick et al., 2001; Wu et al., 2018). Another emerging oomycete infecting pea roots is *Phytophthora pisi*, which was first shown to cause root disease in pea in Sweden. Disease symptoms in pea are similar between the two oomycete pathogens, but symptoms of *P. pisi* are rarely observed on the epicotyl (Heyman et al., 2013). Furthermore, oospores of *P. pisi* can be morphologically differentiated from *A. euteiches* oospores under the microscope (Heyman et al., 2013). Differences in virulence among *A. euteiches* strains are observed in controlled infection experiments (Quillévéré-Hamard et al., 2018; Kälin et al., 2022) but prove difficult for the prediction of cultivar performance in the field where soil microbial compositions are complex (Wille et al., 2020).

Agro-ecological factors have been shown to influence soil microbial abundance and community composition in other legume crops (Naseri and Ansari Hamadani, 2017). The co-occurrence of several pathogens in the pea root rot complex (PRRC) has been reported but their interactions remain largely uncharacterized (Baćanović-Šišić et al., 2018; Chatterton et al., 2019). However, the increased susceptibility to single pathogens of the PRRC in presence of other pathogen species has been shown in controlled greenhouse experiments. Using co-inoculation experiments with *A. euteiches* and several *Fusarium* spp., Willsey et al. (2018) reported a disease reinforcement effect in presence of multiple pathogens. Peters and Grau (2002) showed that co-inoculations of pea with a non-pathogenic *F. solani* strain and *A. euteiches* resulted in significantly more severe disease symptoms compared to single infections with *A. euteiches*. Further, other important factors such as the significant effect of sowing date and depth on fusarium wilt development in chickpea cultivars have been shown by Younesi et al. (2020). Historically, breeding for resistance towards aphanomyces root rot has been most successful combining results from plant-pathogen interactions in both growth chambers and field experiments (Moussart et al., 2001; Wicker et al., 2003; Pilet-Nayel et al., 2005; Abdullah et al., 2017).

In Swedish pea production, current control measures against root rot pathogens focus on diagnosis of occurrence in the field and prevention of high pathogen inoculum levels in fields. Soil testing prior to sowing has been a reliable method for the avoidance of highly infested fields and long periods of crop rotation can prevent inoculum accumulation in the soil (Moussart et al., 2009; Moussart et al., 2013). The production of vining peas for quick-freezing are especially challenging since crop production has to be carried out in proximity of factory sites. Breeding for increased resistance against *A. euteiches* remains the most promising approach in disease control. However, sources of partial resistance in pea are scarce, polygenically inherited and largely affected by environmental effects (Hamon et al., 2013; Desgroux et al., 2016; Lavaud et al., 2016). Pea cultivars with complete resistance to aphanomyces root rot are lacking, but several cultivars with partial resistance have been used in breeding programs. Among them, the landrace PI180693 has been identified as a source of resistance towards *A. euteiches* by Lockwood (1960) and has been used in several studies for its potential to tolerate *A. euteiches* infection (Pilet-Nayel et al., 2002; Wicker et al., 2003). Further, PI180693 has shown to maintain high levels of resistance towards fusarium root rot in both controlled and greenhouse conditions (Grünwald et al., 2003; Infantino et al., 2006; Coyne et al., 2019). However, the landrace is associated with unfavorable breeding traits, such as extremely long internode length (long haulm), pale peas, normal leaves and round seeds with a starchy flavor. In modern crop production, semi-leafless and shorter varieties are preferred, as they will remain more erect at harvest, which reduces the risk of picking up small stones and soil particles that can contaminate the produce. Further, peas for quick freezing should have a ‘sweet flavor’ as well as a uniform, bright and attractive green color. Therefore, PI180693’s growth phenotype is unsuitable for commercial cultivation and quick-freezing.

Our study aimed at evaluating the usefulness of the partial resistance against aphanomyces root rot originating from PI180693 in practical pea breeding, with emphasis on disease range and intraspecific pathogen variation, effectiveness and consistency. We used six back-crossed pea lines from a cross between PI180693 and the commercial variety Linnea to investigate (i) variation in disease resistance between breeding lines, (ii) interactive effects between disease resistance and virulence of *A. euteiches* strains, and (iii) the predictive power of climate chamber and greenhouse pot bioassays for estimating pea field performance. We show that the partial resistance towards aphanomyces root rot derived from PI180693 is useful for applied, commercial breeding and how monitoring the presence and virulence levels of pathogen populations is important for development and deployment of durable root rot resistant cultivars.

## 2 Materials and methods

### 2.1 *Aphanomyces euteiches* cultivation and growth

The *A. euteiches* strains used in this experiment originate from Sweden (SE51 and SE58) and the United Kingdom (UK16). All strains have been used in commercial breeding experiments, as they are known to differ in virulence on pea. Strain SE58 was previously included in a phenotyping assay and shown to be of intermediate virulence. All three strains were described to belong to the same genetic cluster in previous population genetic analyses and were maintained as described in Kälin et al. (2022). Prior to be used as inoculum, strains were grown for two weeks on corn meal agar (CMA, BD Biosciences, San Jose, CA) at 20°C in the dark.

### 2.2 Pea breeding material

Two BC1F8 lines (Z1654-1 and Z1656-1) and four BC2F6 lines (Z1701-1, Z1701-2, Z1707-1 and Z1707-02) were included in this study. These six lines where selected based on screening results of various lines in greenhouse tests (data not shown). The selected lines showed better agronomic performance (yield component parameters and morphology) and tolerance against *A. euteiches* compared to their sibling lines in initial large-scale screenings. The BC1F8 lines were backcrossed once to Linnea, after an initial cross between Linnea and PI180693, whereas BC2F6 lines represents second backcrosses to Linnea in the sixth generation selfed (**Table 1**).

**Table 1:** Information about pea cultivars used in the study.

ID	Type of material	Earliness class*	Leaf type	Flower color	Seed shape
Z1654-1	Breeding line (BC1F8)	+12	semi-leafless	white	wrinkled
Z1656-1	Breeding line (BC1F8)	+12	semi-leafless	white	wrinkled
Z1701-1	Breeding line (BC2F6)	+12	semi-leafless	white	wrinkled
Z1701-2	Breeding line (BC2F6)	+12	semi-leafless	white	wrinkled
Z1707-1	Breeding line (BC2F6)	+12	semi-leafless	white	wrinkled
Z1707-2	Breeding line (BC2F6)	+12	semi-leafless	white	wrinkled
Linnea	Commercial variety (used for BC)	+12	semi-leafless	white	wrinkled
PI180693	Landrace (source of resistance)	+12	leaved	pink	Non-wrinkled

*Earliness class indicated the number of days the cultivar is delayed in green pea harvest relative to reference variety ‘Cabree’ (earliness class 0). BC, backcross number; F, selfing cycle.

### 2.3 Growth chamber and greenhouse assays and phenotyping

Seed surface sterilization was performed following the protocol described in (Kälin et al., 2022) with minor changes. Square plastic pots (0,254 l) were filled with a first layer of vermiculite (Sibelco, Antwerpen, Belgium), on which an agar plate discs (8,5 cm diameter) with *A. euteiches* mycelium were placed in all pathogen treatments. For the infections, only plates fully covered with mycelia were used. The pots were then filled up with vermiculite in which five holes (3 cm depth, 1 cm diameter) were made to place the sterilized seeds. Tools used for the inoculation of *A. euteiches* were sterilized with 70% ethanol between inoculations, to prevent cross-contamination. Pots inoculated with one *A. euteiches* strain were kept together on a separate tray throughout the incubation in the growth chamber (CMP6050, Conviron) at 22°C, 55% humidity and 150 μmol light intensity in a 12 h light, 12 h dark cycle. Uninoculated pots of each cultivar were used as controls. For maintaining optimal pathogen growth conditions, the trays were filled with 2 cm of water and randomly moved within the chamber to account for uneven light or humidity conditions. The experiment was conducted with five pots with five plants each (biological and technical replicates, respectively). Disease scoring was done after three weeks of incubation and root disease symptoms were graded on a scale from 0 (completely healthy) to 100 (completely dead), by two different persons for every plant and then averaged on pot level. Assays in the greenhouse followed the same protocol but with 10 seeds per pot, five replicates, and 16h light, 8h dark cycle at 20°C and 19°C, respectively. For root dry weight measurements, all roots were harvested per biological replicate (pot) and dried over two days at 60°C before weighing on a Precisa 360 ES (growth chamber trials) or Mettler AT261 Delta Range scale (greenhouse trials).

### 2.4 Field trials and phenotyping

In 2020, two field trials were sown on the 2^nd^ of April (Z20EA) and on the 5^th^ of May (Z20EB) in randomized 1 m^2^ plots (two blocks), whereas a single trial in 2022 was sown on the 23^rd^ of March (R-22-10-91) in randomized 12 m^2^ plots (4 blocks). All trials were conducted in southern Sweden (Skåne) and the choice of fields was made based on information from biotest indicating moderate infection rate by *A euteiches*. The soil biotest test prior season showed disease index 34 for Z20EA, disease index 76 for Z20EB and disease index 36 for R-22-10-91 trials. At the location for Z20EB both *A. euteiches* and *P. pisi* were detected, see **Supplementary Table 1** for field coordinates and soil test scores. For phenotyping, ten plants from each plot were taken to rate the infection on roots and provide a disease index score based on root discoloration, between 0 (completely healthy) to 100 (completely dead). The field Z20EA was scored on the 1^st^ of July 2020, Z20EB on the 7^th^ of July 2020 and field R-22-10-91 on the 7^th^ of June 2022, just before flowering to avoid root darkening due to natural maturation processes. Plant emergence was recorded as the percentage of emerged plants in relation to sowed plants in both field trials in 2020 and as the absolute number of emerged plants per square meter in the 2022 field trial. In field R-22-10-91, plant height, yield (at TR100, kg/ha) and the ratio of green peas compared to the total plant biomass as well as additional growth parameters were measured.

### 2.5 Statistical analyses

In the growth chamber experiment, all disease score values were treated as an average of the disease score values scored by the two scorers. Data were tested for normality and mock scores were excluded from further analyses to approach normal distribution. Two two-way analyses of variance (ANOVA) in R using the aov function (package stats ver. 4.1.0, R Core Team, 2021) were performed to assess the effects of the two factors cultivar and strain on disease index and root dry weight, including the factor’s interactions. Data on root dry weight of uninfected plants was assessed separately using Fisher LSD test on one-way ANOVA residuals. For the analysis of greenhouse trials, we used one-way ANOVAs for disease index and root dry weight including cultivar as independent variable, with Fisher LSD *post-hoc* tests. The correlation coefficient for disease index and root dry weight in the growth chamber trials, and for disease index and germination in the field trials, was calculated using Pearson correlation for normal distributions in R (cor.test function). Field data was analyzed separately for each field. For 2020 fields, one-way ANOVAs on the interaction of disease index and emergence with cultivar were performed and Fisher LSD test was used for mean comparisons between groups. For the 2022 field trial, we performed a two-way ANOVA on disease index including cultivar and block effect and one-way ANOVAs were performed for the breeding traits. The correlations of yield with disease index and emergence for each cultivar were analyzed using linear regression modelling.

### 2.6 Climate data

For the duration of the 2020 field trials, data on temperature, rainfall and relative humidity were retrieved from the closest weather station (56°03’04” N, 12°76’28” E), publicly available on https://www.smhi.se/data/meteorologi/ladda-ner-meteorologiska-observationer. For the 2022 field trial, average air temperature, precipitation (rain) and relative humidity were measured using a mobile weather station installed next to the field (56°01’07.8”N 12°58’16.1”E). In both cases, daily measurements were retrieved and the averages over two weeks were calculated and used in **Supplementary Figure 3**.

## 3 Results

### 3.1 Disease resistance in growth chamber trials

**Figure 1:**
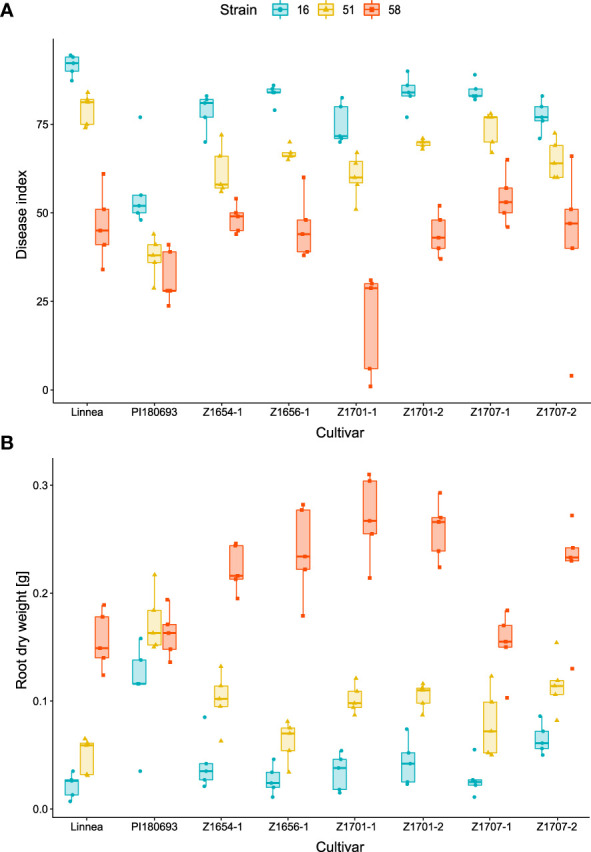
Virulence of *Aphanomyces euteiches* strains on pea cultivars. Disease indices **(A)** and root dry weight measurements **(B)** were assessed in growth chamber trials including six pea breeding lines and the two parental lines upon infection with *A. euteiches* strains UK16 (high virulence), SE51 (intermediate virulence) and SE58 (low virulence). Disease index scores (0 = completely healthy plant, 100 = completely diseased) and root dry weight measurements [g] are averages of five biological replicates.

The growth chamber pot assay showed significant effects of strain (*p* < 0.001), cultivar (*p* < 0.001) and their interaction (*p* < 0.01), on disease index (**Table 2**). *A. euteiches* strains differed in virulence with UK16 being most virulent on all lines, SE51 was of intermediate virulence while SE58 was least virulent on all lines (**Figure 1A**). With low pathogen virulence, i.e. infection with SE58, larger variation in disease symptoms between breeding lines was observed, compared with infection with more virulent strains. The disease index of PI180693 was more consistent upon infection with *A. euteiches* strains differing in virulence (**Figure 1A**). Using Fisher LSD test, breeding lines Z1654-1, Z1656-1, Z1701-1, Z1701-2 and Z1707-2 had significantly (*p* < 0.05) lower disease indices than Linnea upon infection with highly virulent strain UK16 (**Supplementary Figure 1A**; **Supplementary Table 2**). In response to intermediate virulence (strain SE51), the same breeding lines were also significantly more resistant than their susceptible parent (**Supplementary Figure 1B**). However, only line Z1701-1 showed significantly lower disease indices than in Linnea upon infection with the lowly virulent strain SE58 (**Supplementary Figure 1C**).

**Table 2:** Results from analyses of variance of growth chamber and field trials.

Factor	Growth chamber*		Field R-22-10-91*						Field Z20EA^#^		Field Z20EB^#^	
	DI ~cultivar	RDW ~cultivar	DI ~cultivar	Emergence ~cultivar	Yield ~cultivar	Pea biomass ~cultivar		Plant length ~cultivar	DI ~cultivar	Emergence ~cultivar	DI ~cultivar	Emergence ~cultivar
Strain	***	***										
Cultivar	***	***	*	**	*	*		***	*	***	*	**
Strain:cultivar	**	***										
Block			**	***	**	***		**				
Cultivar:block			.	x	.	x		x				

*Two-way ANOVA. #One-way ANOVA. DI, disease index; RDW, root dry weight; significance codes: ‘***’ 0.001 ‘**’ 0.01 ‘*’ 0.05 ‘.’ 0.1 ‘x’ 1.

#### 3.1.1 Root dry weight in growth chamber trials

We measured lowest root dry weight in cultivars infected with the most virulent *A. euteiches* strain UK16 and highest root dry weight in roots of cultivars infected with the SE58 low virulent *A. euteiches* strain (**Figure 1B**). In PI180693, however, the root dry weight was highest in plants infected with SE51 and the difference in root dry weight between roots infected with the three strains was lower compared to other cultivars. Both *A. euteiches* strains and pea cultivars, as well as their interaction, showed to have a highly significant (*p* < 0.001) effect on root dry weight in the growth chamber pot trials (**Table 2**). Fisher LSD tests on cultivar comparisons revealed that upon infection with highly virulent strain UK16, only line Z1707-2 had significantly higher root dry weight than Linnea (**Supplementary Figure 2A**; **Supplementary Table 2**). In response to intermediate virulence (strain SE51), breeding lines Z1654-1, Z1701-1, Z1701-2, Z1707-1 and Z1707-2 scored significantly higher root dry weight than the susceptible parent (**Supplementary Figure 2B**). The same breeding lines, with exception of Z1707-1, also scored higher root dry weight upon infection with the lowly virulent strain SE58, including line Z1656-1 (**Supplementary Figure 2C**)

Root dry weight measurements of the non-inoculated controls showed natural variation in root volume between cultivars. With an average root dry weight of 0.36 g per biological replicate, breeding line Z1654-1 showed to have non-significantly (*p* > 0.05) lower root dry weight scores than PI180693 (average 0.396g) whereas dried roots of line Z1707-1 did not differ from Linnea (0.237g and 0.19g, respectively). All other breeding lines had intermediate root dry weight scores compared to their parent cultivars (**Table 3**).

**Table 3:** Root dry weight of uninfected pea cultivars in growth chamber experiments.

Cultivar	Root dry weight [g]*	Standard deviation	Fisher LSD^#^
Linnea	0.1894	0.04159086	e
PI180693	0.3962	0.07156256	a
Z1654-1	0.3598	0.03089822	ab
Z1656-1	0.3314	0.03415845	b
Z1701-1	0.3280	0.03205464	bc
Z1701-2	0.2698	0.04702871	cd
Z1707-1	0.2372	0.02060825	de
Z1707-2	0.2714	0.06148008	cd

*Roots were harvested after three weeks, and root dry weight values correspond to the average across five biological replicates (pots) with five plants each. ^#^Fisher LSD test was applied on one-way ANOVA residuals. Letters a-e indicate significant (p < 0.05) different between group means.

### 3.2 Disease resistance and root dry weight in greenhouse trials

**Figure 2:**
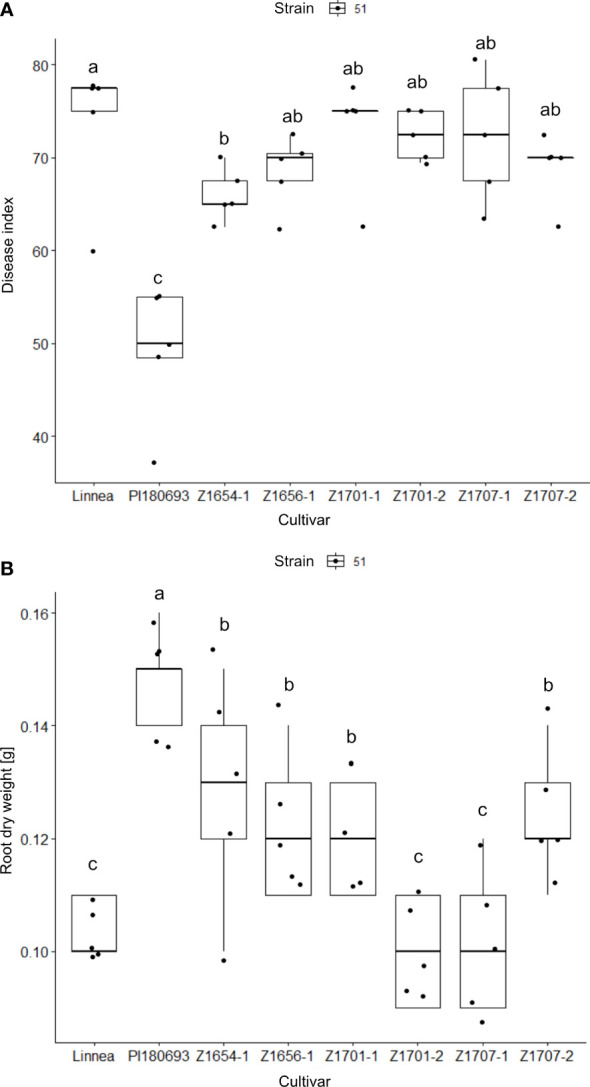
Disease index and root dry weight measurements in greenhouse trials. Disease index scores **(A)** and root dry weight measurements **(B)** in greenhouse trials, including six breeding lines and the two parental lines, upon infection with *A. euteiches* strain SE51 with intermediate virulence Disease index scores (0 = completely healthy plant, 100 = completely diseased) and root dry weight measurements [g] are averages of five biological replicates. Letters a-c indicate Fisher’s significant (*p* > 0.05) differences between means of disease indexes and root dry weight between cultivars.

The effect of cultivar on measured disease indices showed to be highly significant (*p* < 0.001) in the greenhouse trials (**Table 2**). Fisher LSD tests on the ANOVA results showed that only breeding line Z1654-1 was significantly (*p* < 0.05) more resistant than Linnea upon infection with the intermediately virulent *A. euteiches* strain SE51 (**Figure 2A**). The effect on root dry weight was also highly significant (*p* < 0.001, **Table 2**). PI180693 displayed the highest root dry weight, whereas root dry weights of breeding lines Z1656-1, Z1701-1, Z1654-1 and Z1707-2 were significantly (*p* < 0.05) higher than Linnea and lower than PI180693 (**Figure 2B**).

### 3.3 Disease resistance and plant emergence in 2020 field trials

*A. euteiches* oospores were identified microscopically in fields Z20EA and Z20EB. In field Z20EB, *P. pisi* was also detected in soil tests and disease indices were higher on average. During the 2020 field seasons, air temperatures and relative humidity were lower than in year 2022 (**Supplementary Figure 3**).

**Figure 3:**
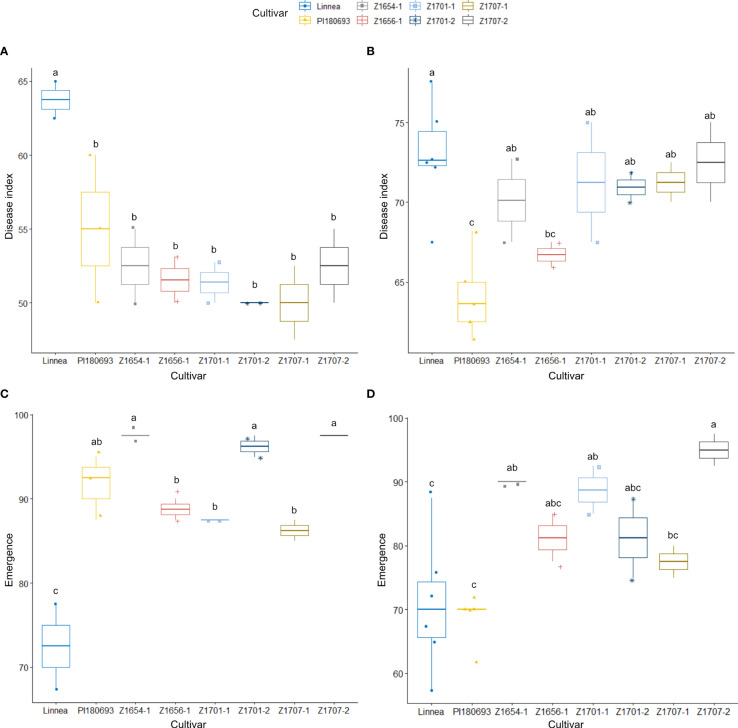
Disease index scores and emergence rates in 2020 field trials. Disease index scores **(A)** and emergence rates **(C)** for field Z20EA and field Z20EB with co-occuring *P. pisi*, **(B**, **D)**, respectively, are averages of two replicates for every breeding line and additional replicates for PI180693 and Linnea. Disease index is measured on a scale from 0 (completely healthy plant) to 100 (completely diseased) and emergence levels indicate the percentage of plants emerged compared to seeds sown. Letters a-c indicate Fisher’s significant (*p* > 0.05) differences between means of disease indexes and emergence rates.

In field Z20EA, Linnea was the most susceptible genotype with a significantly (*p* < 0.05) higher disease index compared with PI180693 and all breeding lines (**Figure 3A**). There were no differences in disease index between PI180693 and breeding lines. There was also a significant (*p* < 0.001) cultivar-effect on emergence in field Z20EA (**Table 2**), where Linnea showed a lower (*p* < 0.05) emergence compared with PI180693 and all breeding lines (**Figure 3C**). Disease index and emergence were significantly negatively correlated in field Z20EA (Pearson R = -0.637, *p* < 0.01).

In field Z20EB, where *P. pisi* co-occurred with *A. euteiches*, cultivar Linnea displayed the highest disease index, while PI180693 had the lowest (*p* < 0.05, **Figure 3B**). Only breeding line Z1656-1 had significantly (*p* < 0.05) lower disease index compared with Linnea (**Figure 3B**). Seedling emergence was significantly (*p* < 0.05) higher in breeding lines Z1707-2, Z1654-1 and Z1701-1 compared with Linnea (**Figure 3D**). Interestingly, no difference in seedling emergence was observed between PI180693 and Linnea (**Figure 3D**). Unlike in field Z20EA, there was no correlation between disease index and emergence in field Z20EB (Pearson R = 0.331, *p* > 0.05).

### 3.4 Disease resistance and plant emergence in 2022 field trial

**Figure 4:**
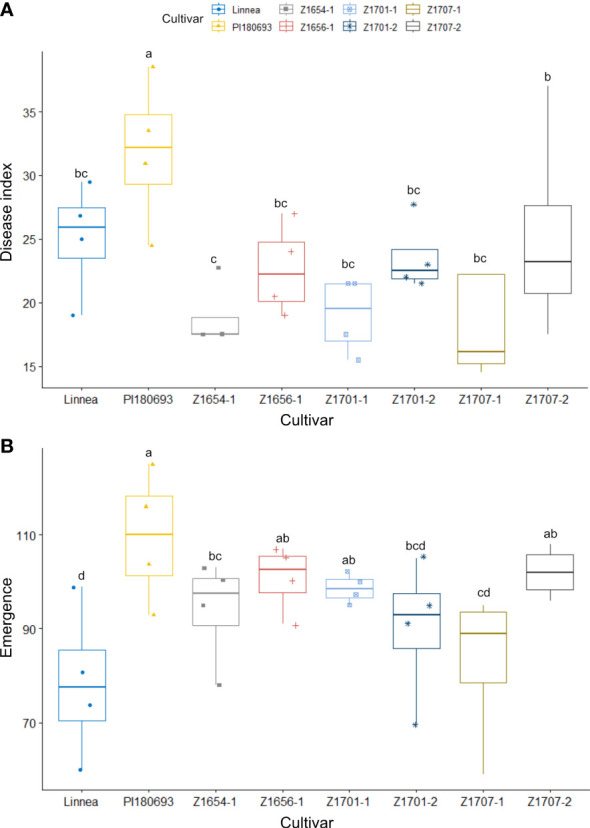
Disease index scores and emergence rates in the 2022 field trial. Disease index scores **(A)** and emergence rates **(B)** in field R-22-10-91. are averages of four replicates per cultivar. Disease index is measured on a scale from 0 (completely healthy plant) to 100 (completely diseased) and emergence levels indicate the percentage of plants emerged compared to seeds sown. Letters a-c indicate Fisher’s significant (*p* > 0.05) differences between means of disease indexes and emergence rates.

As plots in field R-22-10-91 were larger than in fields Z20EA and Z20EB, we analyzed the effect of block size in our two-way ANOVA. Both cultivar and block had a significant effect on disease index (*p* < 0.05 and *p* < 0.01). The interaction effect of block and cultivar was not significant (*p* > 0.1, **Table 2**). Overall disease indices in field R-22-10-91 were lower compared with measured disease severity in the 2020 field trials but warmer average air temperature, less precipitation and higher relative humidity, especially during the sowing period, were measured in the 2022 field season (**Supplementary Figure 3**). Surprisingly, PI180693 scored the highest average disease index compared to all other cultivars (*p* < 0.05). Fisher comparisons between means of disease index per cultivar showed that no breeding line was significantly (*p* < 0.05) more resistant than the susceptible parent Linnea (**Figure 4A**).

Both cultivar and block had a significant effect on seedling emergence in field R-22-10-91 (*p* < 0.01 and *p* < 0.001, respectively, **Table 2**). Seedling emergence was significantly (*p* < 0.5) higher in PI180693 and breeding lines Z1707-2, Z1656-1, Z1701-1 and Z1654-1 than in Linnea (**Figure 4B**). In field R-22-10-91, the correlation between disease index and emergence was non-significantly negative (Pearson R = -0.308, *p* > 0.05).

#### 3.4.1 Yield

**Figure 5:**
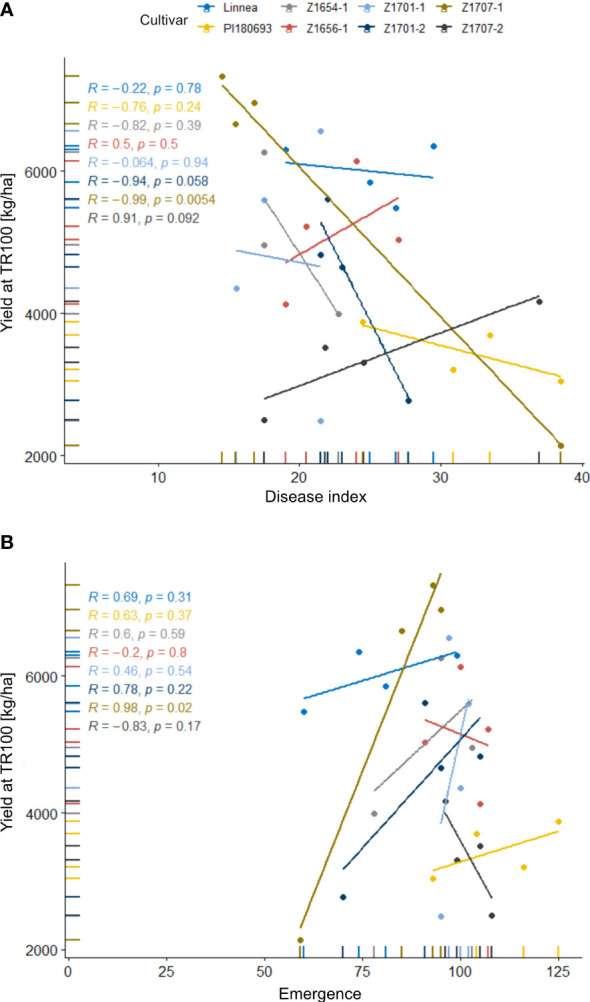
Correlations of yield with disease index scores and emergence rates in the 2022 field trial. Disease index scores **(A)** and yield and emergence rates **(B)** in field R-22-10-91are averages of four replicates per cultivar. Disease index is measured on a scale from 0 (completely healthy plant) to 100 (completely diseased) and emergence levels indicate the number of emerged plants per square meter. Lines represent linear regressions between the two factors, R values show Pearson correlation coefficients.

In field R-22-10-91, block had a significant (*p* < 0.01) effect on yield, as well as cultivar (*p* < 0.05, **Table 2**). Breeding lines Z1701-2 and Z1707-2 had significantly (*p* < 0.05) lower yields than Linnea, but the yield of the other breeding lines did not differ from their commercially used parent. Interestingly, disease indices of lines Z1656-1 and Z1707-2 correlated positively with yield while all other cultivars showed a negative correlation (**Figure 5A**). The same two breeding lines also showed positive correlations between yield and emergence in linear regression analyses (**Figure 5B**).

#### 3.4.2 Percentage of green peas compared to total plant biomass

Both cultivar and block had a significant (*p* ≤ 0.05) effect on the amount of green peas per total plant biomass in field R-22-10-91 (**Table 2**). The percentage of peas versus total plant biomass in breeding lines Z1701-1 (17.7%) and Z1654-1 (17.3%) did not differ compared to 14.1% in Linnea (**Supplementary Table 3**). Interestingly, there was no correlation between disease index and the amount of peas versus the total plant biomass (Pearson correlation coefficient, R = 0.23, *p* > 0.05).

#### 3.4.3 Plant height

In field R-22-10-91, both cultivar and block had a significant (*p* < 0.001 and *p* < 0.01, respectively) effect on the average plant height (**Table 2**). Cultivar PI180693 grew the tallest with an average plant length of 151 cm (**Supplementary Table 3**). The average length of other breeding lines was comparable to Linnea, except lines Z1654-1 and Z1656-1 that grew significantly (*p* < 0.05) taller than Linnea with average plant lengths of 77.6 cm and 81.8 cm.

#### 3.4.4 Number of pods per plant and average length of second node pod

In the 2022 field trial, the number of pods per plant as well as the length of the second node pod were measured and compared to the Linnea phenotype. Breeding line Z1707-2 had significantly less (*p* < 0.05, average 5.88) pods per plant than Linnea (average 7.5) while line Z1654-1 had more with an average of 9.12 (**Supplementary Table 3**). Comparing the average lengths of second node pods, breeding lines Z1656-1, Z1701-1 and Z1654-1 did not differ from the Linnea phenotype with an average length of 56.6 mm while the other breeding lines were comparable to the PI180693 phenotype with an average of 43.8 mm, (**Supplementary Table 3**).

## 4 Discussion

Taken together, our results show that the resistance from PI180693 can successfully be deployed in pea breeding line crosses. We found that some breeding lines are more resistant than their susceptible parent Linnea in field conditions and in growth chamber trials at low pathogen virulence levels. Line Z1654-1 scored lowest disease index on average (11.5% lower than Linnea) in both controlled experiments and scored on average 42% higher in root dry weight measurements compared to the susceptible parent. At lower pathogen pressure, line Z1701-1 showed to be significantly more resistant than both parents in the growth chamber trials with a 58.5% lower disease index than Linnea and 39.5% lower than PI180693. Interestingly, measured disease indices of PI180693 varied less in response to different virulence levels of *A. euteiches* compared with the breeding lines, indicating that the original source of resistance in PI180693 is more robust to varying pathogen virulence levels and partially lost during the breeding steps. This emphasizes the polygenic nature of the resistance and indicates that allele combinations for optimal disease resistance is yet to be achieved in the breeding lines. Along with this, we observed a negative correlation between pea root dry weight and disease index upon infections with *A. euteiches* across cultivars. Resistance QTLs in pea have previously been shown to be correlated with increased root volume and architecture (Desgroux et al., 2018). However, it remains to be investigated at which developmental stage the formation of roots is either fully inhibited or drastically reduced.

In our field experiments, the measured disease indices represented the overall plant health, including both root and shoot phenotype, and cannot be directly compared to disease indices in controlled conditions. Soil testing in fields Z20EA and Z20EB confirmed the presence of *A. euteiches* in the soil and in the latter the co-occurrence of *P. pisi.* We observed higher disease indices in field Z20EB compared to field Z20EA, indicating that presence of *P. pisi* enhanced disease levels. Comparing breeding line performance in field Z20EB, we did not find any indication that resistance in PI180693 is active against *P. pisi* infection. Whereas the genetic resistance in pea towards fusarium root rot caused by *Fusarium solani* f. sp. *phaseoli* is known to be inherited quantitatively (Mukankusi et al., 2011), little is yet known about the genes underlying the resistance to the emerging pathogen *P. pisi* (Heyman et al., 2013; Hosseini et al., 2014). In order to be able to make clearer predictions about the performance of the breeding lines upon infection with *P. pisi*, it will be essential to isolate virulent pathogen strains, and perform controlled single infections with the pathogen.

In field Z20EA where only *A. euteiches* was detected, all breeding lines had significantly higher emergence rates than Linnea, whereas in co-occurrence with *P. pisi* (field Z20EB), emergence rates were lower. We hypothesize that the additional presence of *P. pisi*, could have growth inhibiting effects in early plant growth stages and affect seed germination. When assessing emergence rates, natural variation in seed coat morphology must be taken into account, as for example PI180693 has shown to have a harder seed coat in seed germination tests (data not shown). In previous experiments we used pre-germinated pea seedlings that were able to germinate without pathogen pressure (Kälin et al., 2022). In these greenhouse and growth chamber trials we tried to spatially separate the inoculum from the seed, enabling the seeds to also germinate without pathogen pressure. In field conditions, however, seeds are subjected to *A. euteiches* and other root rot causing pathogens from the moment of sowing, which can lead to lower emergence rates. This emphasizes the importance of optimal timing of sowing within a growing season to reduce root rot disease in legume production (Nazer Kakhki et al., 2022).

In our 2022 field trial design, the size of blocks showed to have a significant effect on all analyzed parameters, which also corresponds to the typical patchy occurrence of *A. euteiches* in agricultural fields. Remarkably, PI180693 scored both highest disease indices and emergence rates in field R-10-22-91. None of the breeding lines showed disease index values that were significantly different from Linnea in this field trial, but four lines showed higher emergence rates than their susceptible parent. However, the 2022 season was very different compared with 2020, with moist soil conditions during sowing, followed by a very dry field season with high temperatures and low precipitation that were not conducive for root rot disease. It is known that levels of high soil moisture, due to heavy precipitation, poor drainage or high soil compaction, favor disease development in *A. euteiches* infections (Grath and Håkansson, 1992; Allmaras et al., 2003; Karppinen et al., 2020) and could therefore explain the observed patterns of lower average disease indices in field R-22-10-91, combined with a significant variation in emergence between cultivars.

With exception of two breeding lines, higher disease indices in field R-22-10-91 were associated with lower yield whereas four out of six breeding lines did not differ in yield compared to Linnea. Two of them (Z1701-1 and Z1654-1) were also comparable to Linnea in the ratio of green peas versus total plant biomass and average length of second node pod. Line Z1654-1 even scored more pods per plant than Linnea but inherited PI180693’s tall growth phenotype. Our results confirm how breeding for robust resistance in pea is facing major challenges as resistance towards root rot is polygenically inherited and often associated with unfavorable breeding traits. Positive and negative associations between alleles controlling plant morphological traits, and resistance, suggesting pleiotropic genes involved in underlying resistance QTLs (Poland et al., 2009; Hamon et al., 2013). Desgroux et al. (2016) have reported a broken linkage between the traits of flower coloration and disease resistance against root rot in pea and recommend finer mapping techniques in future resistance breeding.

Our results further highlight the difficulty of predicting breeding line performance in the field based on results from experiments in controlled environments. In growth chamber experiments pressure from other pathogens is removed and only single or controlled co-infections at known virulence levels are assessed. In field conditions, however, the plants are exposed to a variety of PRRC pathogens with potential synergistic or antagonistic effects, as well as to a variety of other microbes (Wille et al., 2020). In summary, we showed the potential use of combining PI180693 partial resistance against aphanomyces root rot with commercially favorable breeding traits in commercial breeding programs.

## Data availability statement

The raw data supporting the conclusions of this article will be made available by the authors, without undue reservation.

## Author contributions

CK, MK, MD, AKB and ME planned and designed, and CK and MD carried out the growth chamber experiments. Field experiments were planned and performed by AKB and A-KA. Data analysis was done by CK and MK. All authors contributed to the article and approved the submitted version.

## Supplementary material

Supplementary Table 2 Combined Fisher LSD test results for growth chamber, greenhouse and field trials.
