# Supplementary material for: Evaluation of pea genotype PI180693 partial resistance towards aphanomyces root rot in commercial pea breeding
Source: Front Plant Sci. 2023 Mar 14;14:1114408. doi: 10.3389/fpls.2023.1114408 (PMC10043495; doi:10.3389/fpls.2023.1114408)
Supplement: Supplementary file 2 [file Table_1.docx]

**Supplementary Table 1: Trial field location, infection level and timing for trail sowing, disease scoring and pea harvesting**

| Year | Trial ID | Trial location | Soil biotest scores prior trial* | Sowing date | Disease rating date | Green pea harvesting date |
| --- | --- | --- | --- | --- | --- | --- |
| 2020 | Z20EA | 56°00'58.9"N 12°50'53.4"E | 34 | 02.04.2020 | 07.07.2020 | *not harvested* |
| 2020 | Z20EB | 56°03'44.9"N 12°55'01.5"E | 76 | 05.05.2020 | 22-24.07.2020 | *not harvested* |
| 2022 | R-22-10-91 | 56°01'07.8"N 12°58'16.1"E | 36 | 23.03.2022 | 07.06.2022 | 10.07.2022 |

*Soil biotests were conducted in greenhouse prior to growing season and show an average disease index of 30 plats (10 plants per pot), rated as 0 for healthy plants/roots and 100 dead plants
