# Supplementary material for: Evaluation of pea genotype PI180693 partial resistance towards aphanomyces root rot in commercial pea breeding
Source: Front Plant Sci. 2023 Mar 14;14:1114408. doi: 10.3389/fpls.2023.1114408 (PMC10043495; doi:10.3389/fpls.2023.1114408)
Supplement: Supplementary file 3 [file Table_3.docx]

**Supplementary Table 3: Growth parameters of all pea cultivars of the 2022 field trial**

| Cultivar | Average plant height [cm] | | % peas vs. biomass | | Number of pods per plant | | Length of second node pod [mm] | |
| --- | --- | --- | --- | --- | --- | --- | --- | --- |
|  | mean* | sd | mean | sd | mean | sd | mean | sd |
| Linnea | 61.3 | 5.3 | 14.1 | 2.72 | 7.5 | 1.33 | 56.6 | 4.18 |
| PI180693 | 151 | 11.8 | 11.5 | 2.49 | 8.9 | 1.09 | 43.8 | 2.52 |
| Z1654-1 | 77.6 | 8.45 | 17.3 | 2.65 | 9.12 | 2.03 | 55.4 | 4.19 |
| Z1656-1 | 81.8 | 9.25 | 11.4 | 1.71 | 6.7 | 0.5 | 57.3 | 2.34 |
| Z1701-1 | 53.8 | 8.54 | 17.7 | 2.78 | 6.28 | 0.64 | 48.5 | 3.15 |
| Z1701-2 | 56.8 | 10.2 | 12 | 5.88 | 6.45 | 0.98 | 48.5 | 3.43 |
| Z1707-1 | 61.4 | 8.55 | 11.5 | 2.86 | 7.52 | 1.43 | 55.4 | 4.06 |
| Z1707-2 | 56.5 | 11.3 | 11.3 | 7.45 | 5.88 | 1.55 | 46.4 | 6.72 |

*Mean values are averages of four counts
