# Supplementary figures and images for: Evaluation of pea genotype PI180693 partial resistance towards aphanomyces root rot in commercial pea breeding
Source: Front Plant Sci. 2023 Mar 14;14:1114408. doi: 10.3389/fpls.2023.1114408 (PMC10043495; doi:10.3389/fpls.2023.1114408)

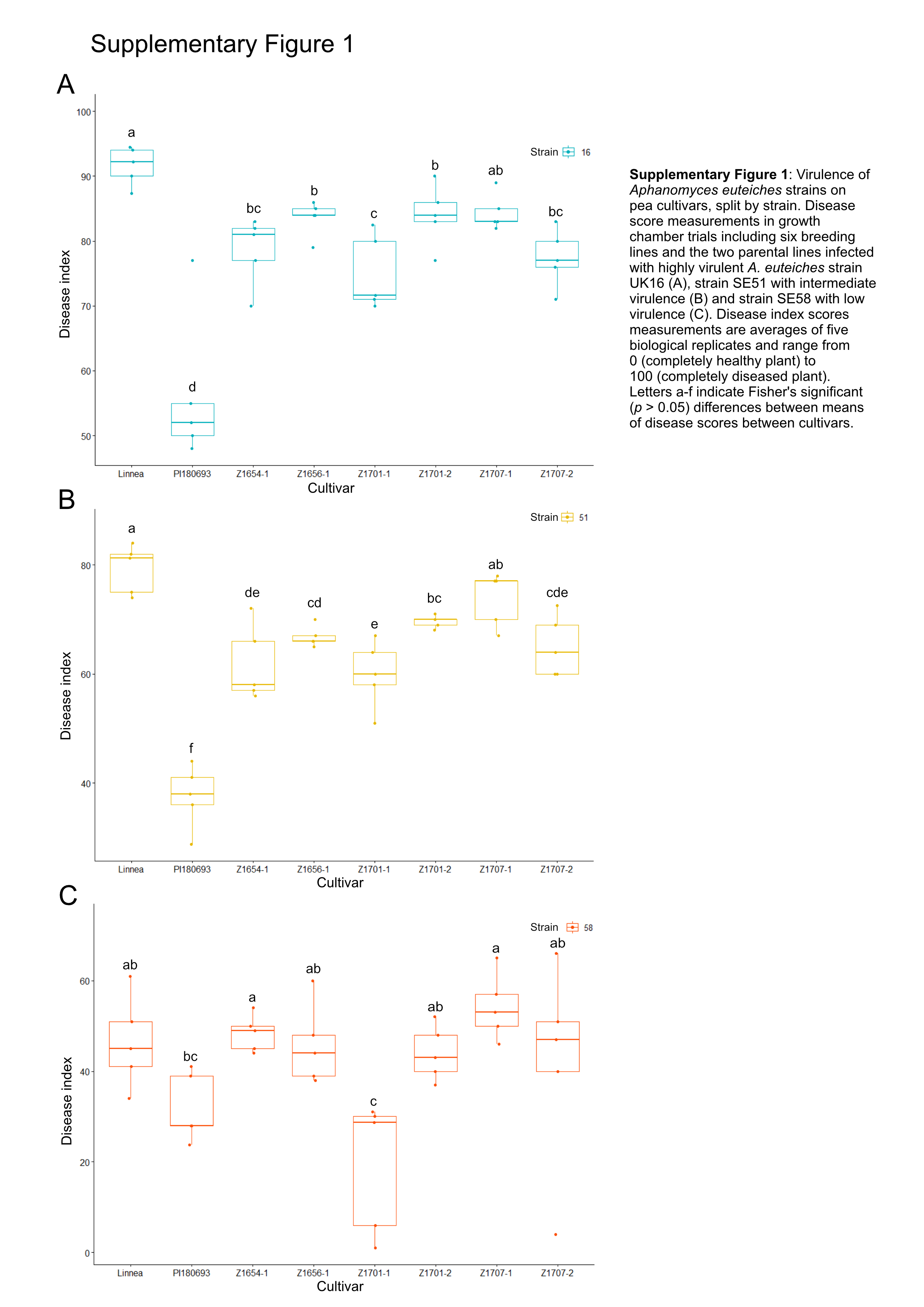

Supplement: Supplementary file 4 [file Image_1.tiff]

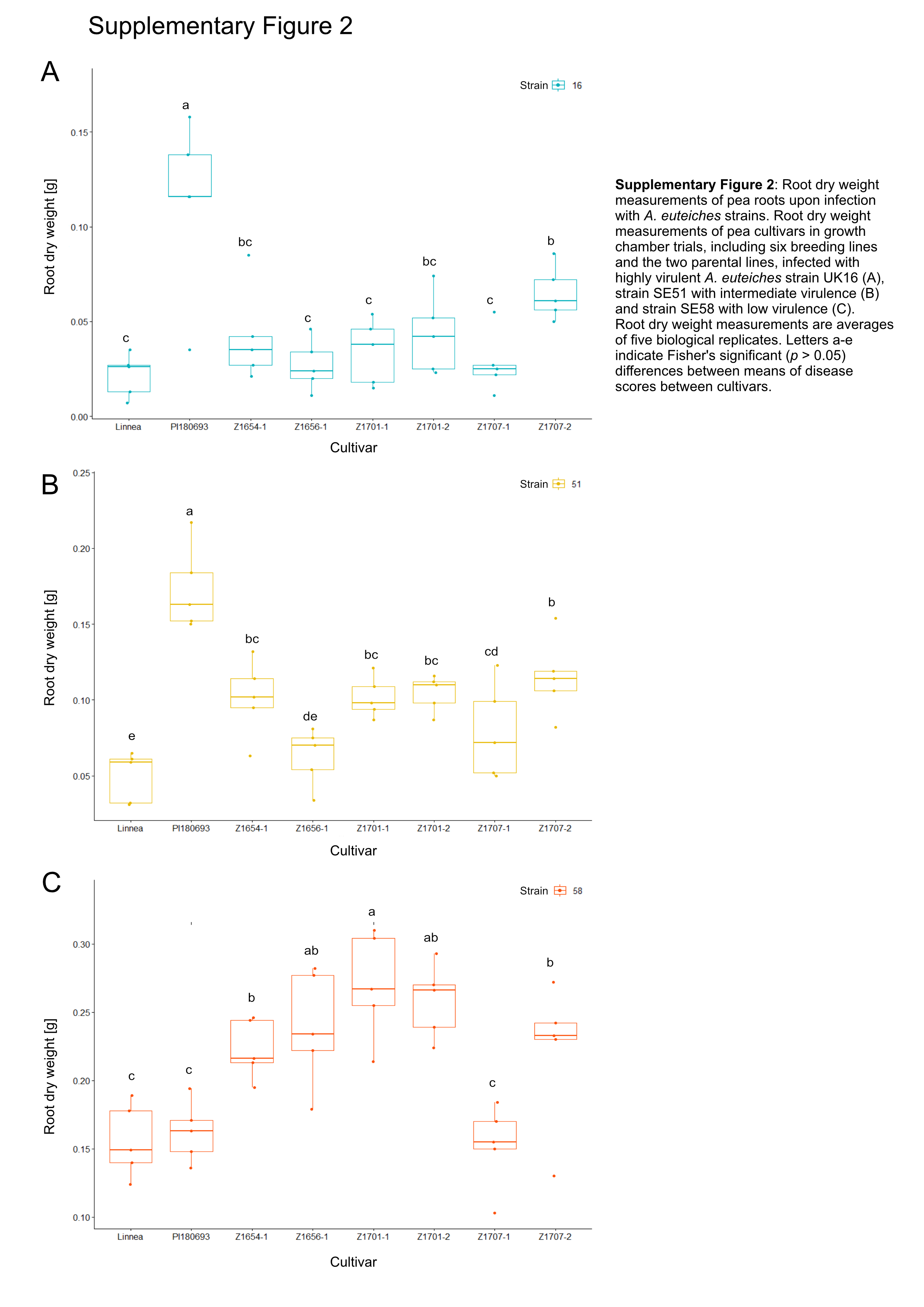

Supplement: Supplementary file 5 [file Image_2.tiff]

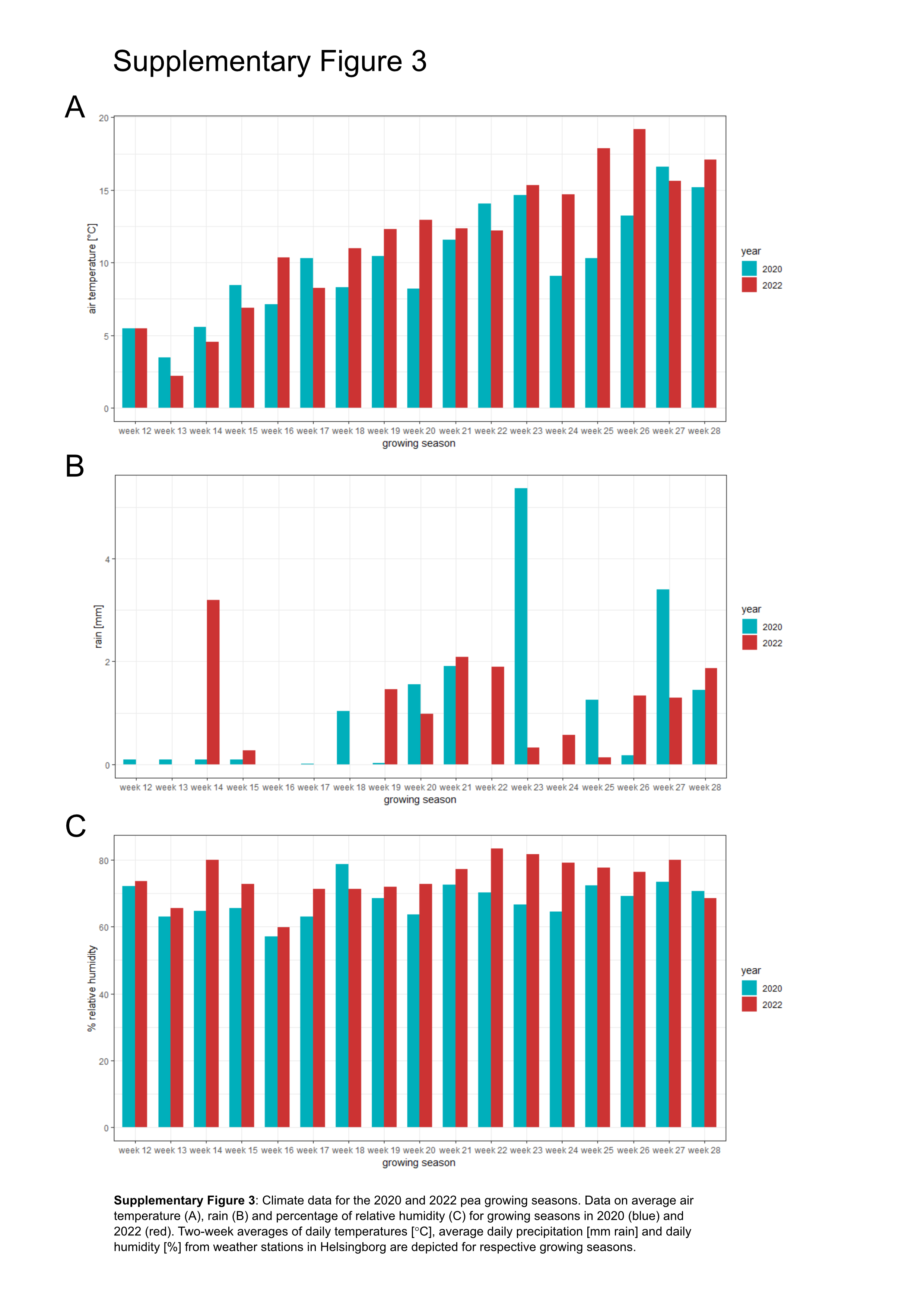

Supplement: Supplementary file 6 [file Image_3.tiff]
